# Evaluation of the Establishment of a Public and Patient Involvement and Engagement Group to Support Clinical Trials in Pakistan: Protocol for a Mixed-Methods Study

**DOI:** 10.12688/wellcomeopenres.23687.1

**Published:** 2025-03-26

**Authors:** Arishay Hussaini, Monaza Khan, Nikhat Ahmed, Madiha Hashmi, Shehla Farooq, Adnan Masood, Srinivas Murthy, Saima Saleem, Zahyd Shuja, Shahnaz Zaman, Arjen M Dondorp, Timo Tolppa

**Affiliations:** 1 Critical Care Research Group, Ziauddin University, Karachi, Sindh, 75000, Pakistan; 2 Patient and Public Involvement and Engagement Group, Ziauddin University, Karachi, Sindh, 75000, Pakistan; 3 The University of British Columbia, Vancouver, British Columbia, Canada; 4 Faculty of Nursing and Midwifery, Ziauddin University, Karachi, Sindh, Pakistan; 5 Centre for Tropical Medicine and Global Health, University of Oxford, Oxford, England, UK

**Keywords:** Patient and Public Involvement, Engagement, Critical Care, Clinical Trial, Low- and Middle-Income Countries, Mixed-Method Study, Co-design

## Abstract

**Background:**

Patient and public involvement and engagement (PPIE) in research is a collaboration between researchers, patients, and the public, enhancing research acceptability, relevance, and impact. There is a growing prevalence of PPIE in high-income country research; however, its integration in low- and middle-income countries (LMICs) remains poorly understood. Recognising this gap, the Ziauddin University Clinical Trials Unit in Karachi, Pakistan, launched a dedicated PPIE initiative in 2022. This study evaluates the engagement process and experiences of patient and public members and researchers to identify barriers and facilitators to participation within the PPIE group.

**Methods:**

The evaluation uses an explanatory sequential mixed-method design. First, the Public and Patient Engagement Evaluation Tool (PPEET) questionnaire will be administered online to group members, coordinators, and senior institutional leads. Insights from questionnaires will be further explored during semi-structured interviews, with questions guided by the Patient Engagement in Research (PEIR) framework, supplemented with analysis of project documentation. Study activities will be conducted in both English and Urdu. The study has been co-designed with PPIE members and is co-led with a public partner. Findings will highlight areas for improvement, inform best practices, and guide the development of more effective engagement strategies.

**Outcome:**

Although focused on a single group, this evaluation lays the groundwork for understanding PPIE practices in LMIC contexts. It provides valuable insights into developing equitable partnerships and improving patient-centred research. This study contributes to a growing body of knowledge, offering practical guidance for implementing PPIE in settings with unique socioeconomic challenges and cultural realities. The findings are expected to benefit the local research community and similar initiatives globally, particularly in regions with comparable challenges.

## Plain language summary

Patient and public involvement and engagement (PPIE) in research is a partnership between researchers, patients, and the community to make health research more relevant and acceptable. This type of partnership allows scientists and doctors to better understand the needs, concerns, and perspectives of the community. Partnering with the community is relatively new in developing countries such as Pakistan, and the best ways to work together are not known. Our team in Karachi launched the first dedicated PPIE group for clinical trials in Pakistan. Through this study, we hope to better understand the experiences of patients, public members and researchers participating in our PPIE group. We will review project documents, and use online questionnaires and interviews to gather data from nine current and past PPIE members, three coordinators, and two senior staff members. The results of this study will help us improve our partnership with patients and the community. We will also use the results to create guidance and supporting materials for others trying to set up PPIE groups in South Asia and elsewhere.

## Introduction

Patient and public involvement and engagement (PPIE) represents a paradigm shift in research by empowering communities to actively shape the generation of evidence in healthcare. The National Institute for Health and Care Research (NIHR) defines it as conducting research “with” or “by” the public, rather than “to”, “about” or “for” them ^1^. By actively involving patients and public partners, and integrating their perspectives, PPIE enhances the relevance, acceptability, and impact of research, aligning it more closely with real-world needs and priorities ^2– 5^. Studies have shown that PPIE delivers ethical and practical benefits, such as influencing study design, improving recruitment by aligning practices with patient needs, guiding the dissemination of results and ensuring greater accessibility of results to the public ^6– 8^.

While PPIE in research has become increasingly prevalent and gained traction in high-research output countries such as Australia, Canada, New Zealand, United Kingdom and the United States, its adoption in low- and middle-income countries (LMICs) remains limited ^9– 12^. A 2022 scoping review found that only one of 33 studies evaluating PPIE processes was conducted in an LMIC ^13^. Reviewing engagement processes in LMICs is essential, as findings from HICs may not directly apply to these contexts with unique challenges, such as structural inequities, social hierarchies, and limited public trust in research ^14^. Furthermore, PPIE efforts in LMICs often rely on community leaders or gatekeepers, whose participation may not always reflect the diverse perspectives within a population. These issues are further compounded by social exclusion, gender inequities, and limited access to healthcare, which all influence how communities engage with research ^15^.

Research also tends to be less prominent and less favourably perceived by the public in many LMICs compared to HICs, making public engagement in research more challenging and likely necessitating different approaches ^16, 17^. Unfortunately, there are no best practice guidelines for PPIE in LMICs due to a lack of dedicated research evaluating effective engagement methods and limited published experiences. This gap highlights the urgent need for studies that explore and develop PPIE strategies specifically for LMICs ^6^.

Recognising the importance of patient engagement in research, the Ziauddin University Clinical Trials Unit (CTU) launched a dedicated long-term PPIE initiative in 2022 ^18^. Supported by an engagement bursary secured by the authors, the initiative aims to promote research acceptability, relevance, and benefits to patients and the public in Karachi, Pakistan. Over its first year, the group has reviewed informed consent processes, proposed key improvements to public-facing materials, and began establishing mechanisms for disseminating trial results to the wider community.

This study aims to evaluate the engagement processes within the PPIE group, explore the experiences of its members, coordinators, and senior institutional leads, and identify barriers and facilitators to effective engagement. By addressing these objectives, the study contributes to the evidence base for PPIE practices in LMICs.

## Methods

### Study design & setting

The study employs an explanatory sequential mixed-method design incorporating quantitative and qualitative methodologies to evaluate patient and public engagement within the PPIE initiative ^19– 22^. Guided by the participatory action research approach, the study was co-designed with the PPIE group members to ensure relevance, inclusivity and alignment with their needs and expectations. The quantitative phase involves administering the validated Public and Patient Engagement Evaluation Tool (PPEET) questionnaire to study participants ^23^. The Public and Patient Engagement Evaluation Tool has been licensed under a Creative Commons Attribution-NonCommercial-Share Alike 4.0 International License. ©2018, Julia Abelson and the PPEET Research-Practice Collaborative. McMaster University. All rights reserved. This process includes standardised administration procedures to maintain consistency, with all participants completing the anonymous survey individually. The tool captures key aspects of engagement and results will inform the qualitative phase, which includes semi-structured interviews and a review of project documentation. Equal weighting will be given to both methods as together they provide a comprehensive understanding of engagement efforts. Findings will be reported using the Guidance for Reporting Involvement of Patients and the Public 2 (GRIPP2) checklist ^24^.

### Study participants

Study participants include current (n=6) and past (n=3) members of the PPIE group, representing both patient and public representatives. The PPIE coordinators (n=3) involved in establishing the group will also be invited to participate. Two senior institutional leads, including a department head and a CTU board representative, will be asked to share their experiences overseeing and integrating PPIE efforts into institutional practices and priorities. Our study aims to include the entire population involved in this initiative to gather a comprehensive understanding of engagement processes across all involved groups i.e. PPIE members, coordinators and senior institutional leads.

### Recruitment

Current PPIE members and coordinators will be introduced to the study during a regular PPIE group meeting or via WhatsApp if unable to attend. Senior institutional leads and past members will be contacted via email. All potential participants will receive the participant information sheet (PIS) in their preferred language (either English or Urdu), outlining the study’s purpose, procedures, and confidentiality measures. Potential participants will have opportunities to ask clarifying questions in person, via WhatsApp or through email. Participants will be given a two-week window to complete the questionnaire, with reminders issued by the study lead after one week and two days before the deadline. Following this, all participants will be invited to participate in semi-structured interviews, regardless of whether they completed the questionnaire. The PIS will be re-shared at this stage.

### Data collection

The study involves three key components for data collection: a validated questionnaire, semi-structured interviews, and project documentation review.

***Public and Patient Engagement Evaluation Tool (PPEET).*** The PPEET questionnaire, a widely used and validated tool developed by researchers at McMaster University, will be used to assess engagement effectiveness on a series of Likert scale items ^23^. Participants will respond to statements on a 5-point Likert scale ranging from “Strongly disagree” to “Strongly agree,” with optional free-text fields allowing respondents to expand on their experiences.

The questionnaire will be administered through an online survey tool (Google Forms) in September 2024; 18 months after the researchers started the PPIE project and one year after the first meeting of the PPIE group.

The PPEET is divided into three questionnaires tailored for different stakeholders: project participants, project coordinators, and organisational leads. These assess experiences and involvement at individual, project, and institutional levels, respectively, while providing valuable insights into the broader PPIE initiative. The online questionnaires are anticipated to take 15–20 minutes to complete.

*Participant Questionnaire* will employ Module B, focused on long-term engagement. The questionnaire comprises 22 questions addressing four key domains: communication and support for participation, opportunities for sharing views, impact and influence of the engagement process, and general reflections.

*Project Questionnaire* includes three modules designed to evaluate different phases of engagement: planning (Module A), implementation (Module B), and impact assessment (Module C). All three modules will be completed by the PPIE coordinators.

*Organisation Questionnaire* evaluates institutional engagement practices, providing insights into policies, participatory culture, collaboration, impact assessment, and organisational reflections. It is designed to identify trends, document improvements, and facilitate audits. This questionnaire will be completed by both senior institutional leads.

Demographic data, such as age, sex, education, ethnicity, and professional status, will not be collected through the questionnaire in order to ensure anonymity. Instead, demographic information previously recorded as part of routine project documentation will be reported. To ensure accessibility, the questionnaire will be translated into Urdu. The translation will be confirmed by the PI and co-PI, who are fluent in both English and Urdu. Prior to distribution, the translated version will be piloted for coherence. Additionally, the PPIE co-lead will be available to clarify terminology or content as needed.

***Semi-structured Interviews.*** All participants will be invited to participate in semi-structured interviews lasting 30–60 minutes, at the university or their preferred location, conducted in English or Urdu based on their preferences. The interview guides are aligned with the Patient Engagement in Research (PEIR) framework ^25^ to explore the perceived benefits of engagement, challenges faced, and opportunities for improvement. Insights from the questionnaire will inform and refine the interview questions. The interview guide for senior institutional leads has been adapted to focus on organisational-level considerations using the PPEET domains, such as policies and strategies for PPIE, as the PEIR framework is not tailored to this context. Interviews will be audio-recorded, transcribed verbatim, and translated into English if conducted in Urdu. Participants will have the opportunity to review and verify their transcripts to ensure accuracy.

The study lead and PPIE Coordinator (AH) will primarily interview researchers, while the co-lead and PPIE member (MK) will focus on interviewing PPIE members to manage any potential power imbalances during the interviews. While one facilitates the interview, the co-lead will observe to ensure consistency and capture additional thoughts. Both leads will interview each other first to mitigate bias in subsequent interviews.

***Project documentation.*** A review of project documentation will be conducted to triangulate findings from interviews and questionnaires ^23^. This document review will follow the completion of questionnaires and interviews to address any information gaps rather than shaping materials iteratively. Key documents include project logs, meeting minutes, debrief notes and member participation profiles recorded using the ‘My Involvement Profile’ template created by ‘Shaping Our Lives’ ^26, 27^. These documents, created at the beginning and throughout the PPIE project, provide further insights into the engagement process within the group and trials and help mitigate recall bias of interview participants ^21^.

This document review will provide insights into member expectations and how they have been met, trends in participation, and the impact of flexible methods such as online and in-person meetings, by examining attendance patterns and other data. Process measures (e.g. number and type of events conducted, number and impact of materials created) from project logs will also be reported. Meeting minutes which document group discussions and debrief notes, as reflective summaries of these conversations will help in understanding evolving engagement patterns. Additionally, we will review the end-of-year funding report provided to the sponsor to gain an overview of the project’s progress and outcomes.

Due to confidentiality reasons, as agreed upon by the members, meeting minutes and debrief notes will not be made publicly available. However, anonymised data, such as attendance percentages and overall project reports will be published.

## Data analysis & management

Responses from the PPEET questionnaires will be imported into Google Sheets and descriptive statistics will be used to summarise responses to the Likert scale questions. Responses to open-ended PPEET questions, transcripts of semi-structured interviews and project documents will be imported into qualitative data analysis software ( NVivo, released in March 2020) for thematic analysis ^26^. Data analysis will occur parallel to the administration of questionnaires and the conduct and transcription of interviews, allowing the interview guide to evolve and guide deeper exploration of emerging themes. The constant comparative method will be used, with comparisons made within and across interviews, with particular attention paid to conflicting accounts and outliers ^27^.

### Patient & public involvement

The study has substantial input from patient and public partners throughout. A PPIE member is a co-PI actively contributing to study design and will be involved in data collection and analysis. A preliminary draft of the protocol was shared with the wider PPIE group for input before finalisation and they will facilitate the dissemination of study findings to the community. This involves developing plain-language summaries, presentations, and other public-facing materials.

### Study timeline & management

The project will run from September 2024 to February 2025. Ethics applications were submitted in July 2024. The PPEET questionnaire will be administered in September and subsequent interviews will be completed by November 2024. Data analysis will be completed by January 2025. The study is led collaboratively by a public partner (MK) and one of the PPIE coordinators (AH), with input from a steering group consisting of other PPIE coordinators (NA, TT) and senior researchers (SM, AMD, MH) associated with the overall PPIE project that bring extensive research experience to ensure appropriateness of study procedures. Authors AH, MK, NA and MH also provide local expertise to ensure the study’s relevance and appropriateness for the Karachi context and oversee local ethical approvals. 

### Informed consent

For the online questionnaire, potential participants will be able to anonymously record their responses, indicating their voluntary consent to partake in the study. For the semi-structured interviews, individual written consent will be sought and documented. The consent form explicitly states that the interview will be audio-recorded and subsequently transcribed verbatim. Participants will have the opportunity to consider each point and indicate their understanding and agreement. The document also includes essential details such as confidentiality measures, participants' right to withdraw at any stage, contact information for the research team, and information about ethical approval.

### Benefits & risks

This study is a formative process evaluation of engagement within our PPIE group and will be used to identify areas for improvement and address any issues. By optimising engagement methods with our members, the group is more likely to work more efficiently, produce higher quality output and be motivated to sustain their involvement over a longer period of time. Improved engagement practices encourage relationship-building between researchers and public representatives, consolidating their commitment to bringing change in the realm of research. The participatory approach of this study fosters collaborative decision-making and builds research capacity in the evolving scientific field of PPIE, particularly for those with limited prior experience. Insights gained may inform similar regions and offer insights to the global research community on best practices for patient and public involvement in LMICs. This is a minimal risk study, aimed to understand experiences of engagement within a PPIE group. The questions are not of a sensitive nature and are not expected to evoke feelings of distress in the participants. The participatory action research approach additionally helps ensure that questions and topics of discussion are acceptable and relevant to participants.

### Participant confidentiality & compensation

All data collected during the study, including questionnaire responses, audio recordings and transcripts, will be stored securely for 10 years on an institutional designated shared drive accessible only to authorised personnel. All members of the PPIE group, both current and former, will be compensated for their time and travel according to the existing compensation policy of the PPIE group. Compensation will not be offered to the coordinators or senior leads who will be interviewed during their regular working hours. The PPIE members will review the protocol, results and manuscripts before finalisation, without access to identifiable study materials.

### Dissemination & publication policy

The study co-leads (AH, MK) will write the first draft of the manuscript, abstract, and any other publications resulting from the study. Our objective is to disseminate our findings effectively across Pakistan, ensuring relevance and accessibility to the target audience. Results will be shared with the Ziauddin University PPIE group through infographics, presenting key findings in both English and Urdu. We will share publication links with researchers in CTUs nationwide, existing PPIE groups and collaborators in the South Asian region interested in establishing or improving PPIE groups along with a toolkit containing useful checklists and documentation (e.g. terms of reference). Additionally, we aim to publish our results in academic journals like BMC Research Involvement & Engagement and present them at academic conferences such as the International Clinical Trial Methodology Conference 2026 and the Oxford Global Health and Bioethics International Conference scheduled for 2025.

## Discussion

This study improves understanding of effective engagement processes, which is not well explored in Pakistan or South Asia. Having a PPIE member as a co-lead ensures that the study design and documentation are appropriate for participants. The use of the validated PPEET tool and a framework-driven evaluation will generate results that are both generalisable and comparable. By incorporating feedback from both current and former participants, the study minimises risk of bias from respondents who might view the initiative more favourably. Moreover, the findings have the potential to make a meaningful impact by informing and improving engagement practices for both researchers and members. Conducting the evaluation after the first year of a long-term initiative is beneficial as most evaluations occur post-project conclusion, when recall bias can affect responses and changes cannot be implemented to improve engagement practices ^20^.

This evaluation is not intended to produce externally valid and broadly generalisable knowledge, but insights from it will likely be useful for others, particularly in South Asia, to inform the development and refinement of PPIE initiatives. It offers a valuable baseline for engagement dynamics at our organisation and lays the groundwork for ongoing monitoring and evaluation by periodically administering the PPEET (i.e. annually), as recommended by the tool’s creators ^23^. Although resource constraints prevented formal back-and-forth translation of the PPEET tool, the translation will be reviewed by two researchers proficient in English and Urdu to ensure accuracy and fidelity.

The study co-leads (AH, MK) and nearly all protocol co-authors are planned participants for this study, in line with the participatory action research approach, introducing a potential response bias. However, the study aims to capture subjective responses. Furthermore, the involvement of senior researchers (ADM, SM), who are non-participants, in the interpretation of findings will mitigate such bias.

## Conclusion

This study is an initial step to explore and understand engagement processes within our patient and public involvement and engagement group, to advance and and improve public representation in research, especially in Pakistan. By utilising a participatory action research approach and robust evaluation methods, we aim to develop meaningful strategies and build capacity for sustained engagement with public partners. The findings can be used to inform local institutional practices, as well as contribute to the limited database on context-specific PPIE engagement in LMICs.

## Ethics and consent

 Minimal risk ethical approval for this study was obtained from the Oxford Tropical Research Ethics Committee (OxTREC) (Reference: 562–24) on 4th September 2024. This approval is valid for the planned duration of the study and is subject to obtaining local ethical approval. Additionally, ethical approval was granted by the Ziauddin University Ethics Review Committee on 30th September 2024 (Reference: 9040824MHCCM).

This study adheres to the ethical principles outlined in the Declaration of Helsinki.

For the online survey, participants will record consent at the start of the survey. An introduction outlines the study’s purpose, procedures, potential risks, and confidentiality measures. Participants are given the option to either continue with the survey if they agree to participate or select “No” to decline participation, which would immediately exit the survey.

For the semi-structured interviews, written informed consent will be obtained from each participant before the interview. The consent form explicitly states that interviews will be audio-recorded, transcribed and anonymised before analysis. Participants will have the opportunity to review and ask clarifying questions before providing consent.

## Data Availability

No underlying data are associated with this article. **Open Science Framework: Evaluation of the Establishment of a Public and Patient Involvement and Engagement Group to Support Clinical Trials in Pakistan,** https://doi.org/10.17605/OSF.IO/VQPZH ^28^. **This project contains the following extended data:** Participant information sheets (in English and Urdu), Consent forms for the semi-structured interview (in English and Urdu), Questionnaires (in English for PPIE Coordinators and Senior Institutional Leads, in English and Urdu for Members), Interview guides for the semi-structured interviews (in English for PPIE Coordinators and Senior Institutional Leads, in English and Urdu for Members), Qualitative study protocol checklist (ObsQual). Data are available under the terms of the Creative Commons Attribution 4.0 International license (CC BY 4.0).

## Software availability

The software used to analyse qualitative data in this study, NVivo, is a proprietary software. QualCoder is a free, open-source alternative that provides similar qualitative analysis functionalities. The Public and Patient Engagement Evaluation Tool has been licensed under a Creative Commons Attribution-NonCommercial-Share Alike 4.0 International License. ©2018, Julia Abelson and the PPEET Research-Practice Collaborative. McMaster University. All rights reserved.
