# Supplementary material for: Evaluation of the Establishment of a Public and Patient Involvement and Engagement Group to Support Clinical Trials in Pakistan: Protocol for a Mixed-Methods Study
Source: Wellcome Open Res. 2025 Dec 15;10:162. Originally published 2025 Mar 26. [Version 2] doi: 10.12688/wellcomeopenres.23687.2 (PMC12744197; doi:10.12688/wellcomeopenres.23687.2)
Supplement: Peer review history

# Reviewer report: Reviewer response for version 2

**DOI:** 10.21956/wellcomeopenres.27674.r142329

**Published:** 2025-12-27

**Authors:** Gwenda Simons

**Affiliations:** 1 University of Birmingham, Birmingham, England, UK

I have reviewed the author response, and I believe that the authors have addressed my concerns and suggestions. I have no further reservations and would like to see the article approved as such. I am excited to see this work moving forward.

Is the study design appropriate for the research question?

Yes

Is the rationale for, and objectives of, the study clearly described?

Yes

Are sufficient details of the methods provided to allow replication by others?

Yes

Are the datasets clearly presented in a useable and accessible format?

Not applicable

Reviewer Expertise:

NA

I confirm that I have read this submission and believe that I have an appropriate level of expertise to confirm that it is of an acceptable scientific standard.

# Reviewer report: Reviewer response for version 1

**DOI:** 10.21956/wellcomeopenres.26131.r127089

**Published:** 2025-09-03

**Authors:** Gwenda Simons

**Affiliations:** 1 University of Birmingham, Birmingham, England, UK

The paper describes the protocol for the evaluation of the establishment of a PPIE group to support clinical trials in Pakistan using a mixed method study.  There is a growing prevalence of PPIE in health research, including clinical trials in high income countries. However, PPIE is less prevalent in low and middle-income countries. The study will use a mixed method design, asking all the stakeholders (PPIE group members, PPIE coordinators and senior institutional leads) to complete the Public and Patient Engagement Evaluation Tool (PPEET) ^1^ at several time points, and conducting semi-structured interviews. In addition, the project documentation will be reviewed. The study will be conducted in two languages widening participation. Critically the study was co-designed with PPIE members and will be co-led by a public partner. 

 This project is timely and worthwhile, and I have read the protocol with great interest. 

**A few remarks regarding the INTRODUCTION  section:**

 *The reference to INVOLVE website is rather old and INVOLVE no longer exists (ref 1). It would be better to for example refer to the Briefing notes for researchers - public involvement in NHS, health and social care research and in particular Briefing note two: what is public involvement in research? ^2^ which contains the same quote. Please update the reference. 

 * I would have liked a little more background on the PPIE initiative that this study is evaluating. I realise that this has been published previously ^3^, but a brief summary here would be welcome.

 *Furthermore, has the PPIE group been involved with any clinical trials and has the composition of the group changed since you set it up? Obviously, this will affect the outcomes of your evaluation exercise, and it would be worth mentioning this in the protocol. 

 * Clarification around what you mean by ‘engagement’ in this context is needed to make the article scientifically sound. There is different terminology used by different people for PPIE. However, engagement often refers to researchers engaging with the public and sharing their findings, whereas ‘Involvement’ tends to refer to involving patients and members of the public in the actual research as research partners, e.g.  when they co-design a study, help write lay summaries etc. From my reading of the current protocol and the terms of reference attached to your previous article, your PPIE group was set up to do more of the latter? See also the NIHR guidelines for PPI ^2^

 *I was wondering how many studies incl. clinical trials conducted in Pakistan mention PPIE in their reports and publications? You refer to the review that only found 33 studies evaluating PPIE in a LMIC ^4^, but surely there are more studies using input from patients and members of the public and describe this in the publications? I realise that this would be another review and I  mainly ask out of interest, but if that information is readily available it might be of interest to the reader?

**A few remarks regarding the METHOD section:**

 *The mixed methodology seems well suited for the purpose of the study and I think you will get rich data from all 3 sections. 

 *However, the sample size will be rather small, even if all PPIE members, both current and past, will take part. Do you think this will affect your study in any way? Please discuss here or in the analysis section. 

 * Would it be an idea to use the PPEET answers of a participant as prompts in their interview? If you were only doing the PPEET and had a bigger sample, I think anonymity would be preferable, but since a) the sample is rather small and b) as they are completing the PPEET and asked to do an interview its likely you would be able to identify them anyway, so perhaps trying to 'combine' the two might be worthwhile?

 *This will be quite time intensive exercise for PPIE members and staff. Are they paid for taking part? 

**A few remarks regarding the DATA ANALYSIS & MANAGEMENT section:**

 *The plans are clearly laid out and I don't have any major concerns. 

**A few remarks regarding the DISCUSSION section:**

 *I believe you have highlighted the main impact of the study as well as any potential restrictions. 

 Some further thoughts:

 *Are there future plans for evaluation of the group? 

 *Are there plans for creating future PPIE groups at other hospitals/ Universities?  

 In Summary, I have read through this protocol with great interest and believe the study is worthwhile. PPIE in clinical trials in LMIC should be fully supported  and effective PPIE groups set up.  A few clarifications are needed as outlined in my report to make this publication and any follow-up publications more impactful and useful for your own and other PPIE groups.

Is the study design appropriate for the research question?

Yes

Is the rationale for, and objectives of, the study clearly described?

Yes

Are sufficient details of the methods provided to allow replication by others?

Yes

Are the datasets clearly presented in a useable and accessible format?

Not applicable

Reviewer Expertise:

Behavioural medicine, rheumatology, patient and public involvement

I confirm that I have read this submission and believe that I have an appropriate level of expertise to confirm that it is of an acceptable scientific standard, however I have significant reservations, as outlined above.

## References

[1] : Supporting quality public and patient engagement in health system organizations: development and usability testing of theP ublic andP atientE ngagementE valuationT ool. *Health Expectations* .2016;19(4) : 10.1111/hex.12378 817-827 10.1111/hex.12378 PMC515271726113295

[2] National Institute for Health and Care Research (2021). Briefing notes for researchers - public involvement in NHS, health and social care research. https://www.nihr.ac.uk/briefing-notes-researchers-public-involvement-nhs-health-and-social-care-research.

[3] : Establishment of a patient and public involvement and engagement group to support clinical trials in Pakistan: Initial lessons learned. *Research Involvement and Engagement* .2024;10(1) : 10.1186/s40900-024-00635-6 10.1186/s40900-024-00635-6 39334505 PMC11429104

[4] : A scoping review of methods to measure and evaluate citizen engagement in health research. *Research Involvement and Engagement* .2022;8(1) : 10.1186/s40900-022-00405-2 10.1186/s40900-022-00405-2 36496455 PMC9737710

# Response

**Published:** 2025-11-21

**Authors:** Arishay Hussaini

**Affiliations:** Critical Care Research Group, Ziauddin University, Karachi, Sindh, Pakistan

We sincerely thank the reviewer for their constructive and encouraging feedback, which has helped strengthen the clarity, structure, and overall coherence of the manuscript. 

 In the Introduction, the reference to the now - discontinued INVOLVE website has been updated to the NIHR Briefing Notes for Researchers (2021). We have also expanded this section to briefly describe the establishment and aims of the Ziauddin University PPIE group, including its contributions to major international clinical trials (REMAP-CAP and Mega-ROX) and the evolution of its membership over time.

 The distinction between “involvement” and “engagement” has been clarified in line with NIHR guidance, noting that our group’s work primarily centres on involvement while also supporting engagement through public-facing activities.

 In the Methods, we clarified that all 14 individuals directly involved with the PPIE group will be included, making this an exhaustive rather than a sampled population. While the sample size is small, this is appropriate for a process evaluation that prioritises depth of understanding over generalisability.

 The description of the PPEET tool has been refined to explain how scores will inform interview prompts, enhancing integration between quantitative and qualitative findings. The section on participant compensation now specifies that PPIE members receive an honorarium and travel reimbursement in line with the group’s policy, while coordinators and institutional leads participate within their regular institutional roles.

 The Discussion has been expanded to outline next steps, including plans for longitudinal evaluation through annual PPEET assessments and exploration of similar PPIE groups at other institutions, contributing towards a potential national PPIE framework in Pakistan.

 Finally, we carefully reviewed the full manuscript to improve flow, reduce redundancy, and ensure consistency in tone, terminology, and reference sequencing.

# Reviewer report: Reviewer response for version 1

**DOI:** 10.21956/wellcomeopenres.26131.r121361

**Published:** 2025-04-22

**Authors:** Michele Andrasik

**Affiliations:** 1 Fred Hutch Cancer Center, Seattle, Washington, USA

What is presented here is a plan for future analysis of a community engagement process.  I would advise the authors to develop a manuscript about the PPIE process focusing on how it was implemented, who was involved, barriers, challenges and factors that facilitated the process.  That would be a great contribution to the literature as there are few examples of this focusing on LMIC in the literature.  I would strongly encourage the authors to submit a manuscript once they have collected and analyzed the qualitative and quantitative evaluation data.  What they have submitted here is a great beginning to the evaluation data manuscript.  In its current state this manuscript is not appropriate for indexing. I want to underscore that this is a really interesting and exciting project, and the authors should definitely publish it. I hope my suggestions for future manuscript submissions are helpful.

Is the study design appropriate for the research question?

Yes

Is the rationale for, and objectives of, the study clearly described?

Yes

Are sufficient details of the methods provided to allow replication by others?

Yes

Are the datasets clearly presented in a useable and accessible format?

Not applicable

Reviewer Expertise:

HIV and TB vaccines, social and structural determinants of health, trauma, mixed methods, community engagement

I confirm that I have read this submission and believe that I have an appropriate level of expertise to confirm that it is of an acceptable scientific standard.
